# Treatment and Rehabilitation Approaches for Stress Fractures in Long-Distance Runners: A Literature Review

**DOI:** 10.7759/cureus.49397

**Published:** 2023-11-25

**Authors:** Spyridon Hadjispyrou, Argyris C Hadjimichael, Angelos Kaspiris, Petros Leptos, Jim D Georgoulis

**Affiliations:** 1 Orthopaedic Department, Children's Hospital "P. A. Kyriakou", Athens, GRC; 2 Orthopaedic Department, Saint Mary's and John's Polyclinic, Nicosia, CYP; 3 Laboratory of Molecular Pharmacology, School of Health Sciences, University of Patras, Patras, GRC; 4 Medical School, University of Nicosia, Nicosia, CYP; 5 First Department of Orthopaedics, Attikon University General Hospital, National and Kapodistrian University of Athens School of Medicine, Athens, GRC

**Keywords:** overuse, exercise, injury, management, marathon, running, bone, stress fracture

## Abstract

Stress fractures (SFs) result from repetitive mechanical stress on bones, leading to an imbalance in osseous tissue adaptation and resulting in cortical fractures. The majority of SFs occur in the lower limb due to excessive mechanical loads. Long-distance runners are highly susceptible to SFs, especially when there is a significant increase in the load or intensity of their activity. Various intrinsic and extrinsic factors contribute to the development of SFs. Common SF locations in long-distance runners include the tibial shaft, femur, metatarsal, and pelvic region. Diagnosis may be delayed due to mild symptoms and unremarkable imaging tests. However, the chronicity and recurrence of misdiagnosed SFs may lead to debilitating complete fractures that are even more challenging to treat. In this review, we present data revealed from published case reports and case series studies obtained through PubMed and Embase databases focusing on the management of SFs in long-distance runners and correlate treatment outcomes with rehabilitation and return to high-level athletic performance.

## Introduction and background

Stress fractures (SFs) result from repeated mechanical stress on normal bones, exceeding their normal remodeling capabilities and leading to microscopic or complete fractures [1]. SFs are often diagnosed in athletes when the frequency or intensity of their physical activity significantly increases, especially in runners with high training workloads [1]. Breithaupt published the first literature report on SF in 1855, describing it as "march fractures" observed mainly as metatarsal fractures in military recruits after long-distance marches [2].

In the majority of cases, SFs occur in the lower extremities, with the tibia being the most commonly affected site, followed by the foot and femur [3]. According to Changstrom et al.'s 2015 epidemiological study, the incidence of SF was 0.8% among 51,773 injuries in high school athletes during 25,268,873 athlete exposures, with an overall SF rate of 1.54 per 100,000 athletes in all sports [4]. Additionally, a study by Tenforde et al. found that over one-fifth of middle and long-distance runners in intercollegiate athletics sustained one or more SF [5].

A history of previously experienced SFs (five times higher) and female gender (2.3 times higher) are significantly associated with a higher risk of lower limb SF in runners compared to the absence of previous SF and male gender, respectively [6]. The female athlete triad - menstrual dysfunction, low energy availability, and decreased bone mineral density - is a strong predisposing factor for SF in female runners [6,7]. Late menarche (onset > 15 years old) is recognized as an intrinsic risk factor, making female runners more prone to SF [8].

Furthermore, when considering extrinsic variables contributing to SF risk, body mass index (BMI) emerges as a significant factor in both genders. Specifically, individuals with a BMI below 19 kg/m^2^ (classified as underweight) or above 25 kg/m^2^ (classified as obese), particularly those with poor muscle development, exhibit an increased risk for SF [2,7,9]. Training load greater than 32 km/week is another extrinsic risk factor doubling or tripling SF probability [5].

The onset of clinical symptoms associated with SFs is usually insidious, with a pre-existing dull pain, focal tenderness, and swelling localized at the point of damage after a training session. Clinical tests, such as the hop test for lower limb SFs and the fulcrum test for femoral SFs, can guide the diagnosis [10]. The differential diagnosis includes shin splints, exertional compartment syndrome, tendinopathy, nerve or artery entrapment syndromes, peripheral nerve injuries, neuropathies, and metatarsalgia [10].

For imaging, a plain X-ray is the initial choice, although findings are often unremarkable. Repeat X-rays after two to three weeks may indicate an occult SF. Advanced imaging, including MRI and bone scintigraphy, is required when symptoms persist and diagnosis is uncertain [11].

In terms of treatment, SFs are categorized into non-invasive therapy and surgical management. Initial conservative treatment (RICE: rest, ice, compression, elevation) with running cessation is effective in most cases, and the majority of patients return to active training within four to six weeks [12]. Surgical management is often reserved for complicated SFs, such as non-union and delayed-union, to prevent complete fractures [10].

The term "long-distance runner" refers to athletes competing in races of 3,000 meters to 42,195 meters [13]. This study focuses on athletes running 21,000 meters and above, covering amateur and professional runners participating in half-marathon and marathon distances.

The aim of this study is to narratively review published literature, including case reports and case series studies, regarding the diagnosis and management of SF in long-distance runners.

## Review

Materials and methods

**Figure 1:**
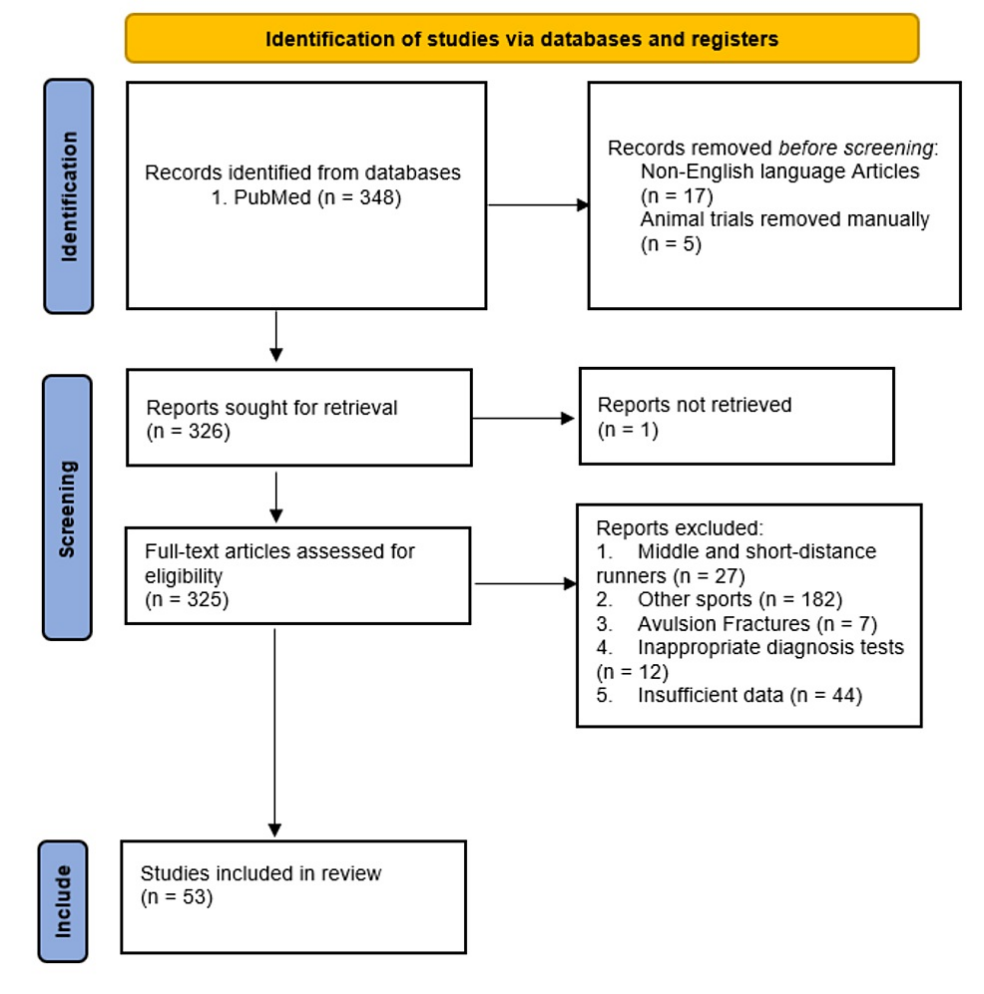
Preferred Reporting Items for Systematic Reviews and Meta-Analyses (PRISMA) flowchart for seeking and identifying included studies.

The authors explored the literature for published case reports and case series studies addressing the management of SFs in long-distance runners. A systematic computer-based literature review search with predefined criteria was performed in the following databases: MEDLINE/PubMed (1946-present) of the National Library of Medicine and Embase (1947-present), as shown in the flow chart (Figure 1). The research methodology used a combination of the following terms: “stress fracture [All fields],” “long-distance [All fields],” “athletes [All fields],” "marathon runners [All fields],” “diagnosis [All fields],” “treatment [All fields],” and "rehabilitation [All fields].” Searching of the reference lists of potentially relevant origin was also performed. The electronic literature search was conducted by the first author S.H. The collected articles were reviewed and examined independently by the authors S.H., A.K., P.L., and J.D.G. Moreover, the senior author A.C.H. independently screened the titles and abstracts to identify relevant studies and extracted significant data evaluating the management of SFs in various bones.

Inclusion Criteria

Inclusion criteria were papers written in English and peer-reviewed journals concerning the management of SFs in long-distance runners. Case reports and case series studies that described the diagnosis, treatment plan, and rehabilitation of SFs in these athletes were included.

Exclusion Criteria

Exclusion criteria were the following: studies considering the management of SFs in non-long-distance runners such as short and middle-distance runners, football players, basketball players, and non-professional athletes; case reports presenting insufficient data about the treatment plan and specific rehabilitation time were also excluded; studies in which SFs were not diagnosed through appropriate imaging tests; avulsion fractures were also excluded from our study.

SFs per anatomic location

Tibia

The tibial shaft is the most common anatomic site for SFs to occur among long-distance runners as a result of repetitive and excessive mechanical loads with approximately 50% of SF cases reported in the literature [14,15]. Overall, tibial SFs are distinguished into two categories, the posterior SF and anterior SF, based on their anatomical location. It has been shown that posterior SFs at the middle to lower one-third of these subtypes are more common and they are associated with a better prognosis [16]. The diagnosis of tibia SFs is very difficult and challenging, including physical examination, X-rays, bone scan, and MRI. Hence, it is recommended that SFs should be primarily included in the differential diagnosis in those runners presenting with vague symptoms and suspicious clinical signs in the knee area, especially when the frequency or intensity of training workouts and running habits have recently increased [17].

Despite tibial SFs being the most common, we only found five case reports in the literature reporting the management and rehabilitation of this type of fracture in long-distance runners [17-21]. All long-distance runners who were diagnosed with tibial shaft SFs were male with a mean age of 25.8 ± 8.2 years old (from 15 to 38 years old) [17-21]. Four out of five cases were complaining of pain, which was initiated during a running session. Three out of five cases were diagnosed with a tibial SF immediately after the onset of symptoms; however, in the remaining two cases, the establishment of diagnosis was delayed approximately three weeks and two months, respectively [17,21]. All reported cases were treated conservatively. According to Bargfeldt et al., the presented long-distance runner in the case report was the only one among the five cases who were followed up, with sufficient fracture healing appearing nine weeks post injury [20]. The rehabilitation period and the time required for a safe return to sport in professional long-distance races were not specified in the rest of the cases.

As a general conclusion from the case reports retrieved, the treatment and rehabilitation of tibial SFs typically involve a conservative approach. This includes a period of relative rest or modified weight-bearing activities to reduce stress on the affected bone. Assistive devices like crutches or walking boots may be used initially to offload the fractured area. Physical therapy interventions and adequate nutrition and supplementation are also important. The duration of rehabilitation and the timeline for a safe return to running vary based on the severity of the fracture and individual factors. Regular monitoring and gradual progression of activities are essential for successful recovery.

Femur

Femoral SFs are categorized based on their anatomical location involving the femoral neck, the femoral shaft, and the condyles [22]. Moreover, the responsible underlying mechanism of femoral SFs was classified by Fullerton and Snowdy as follows: compression, tension, or displaced type [22]. The most common symptom is anterior groin pain and the onset is usually related to intensive active exercising [22]. Although the femur is the 4th most frequent anatomical location for SFs to be developed, a lengthy debate exists regarding the appropriate treatment option required [23].

Based on our literature review, a total of 24 case reports describing long-distance runners’ femoral SFs have been identified [23-46]. The mean age of the patients was 33.2 ± 13.1 years with a range between 15 and 64 years. Fourteen male [25-27,30,33,34,36,37,39,40,42-44,46] and 10 female [23,24,28,29,31,32,35,38,41,45] long-distance runners were diagnosed with a femur SF. Among the 24 identified femur SFs, 20 of them were observed in the femoral neck [23-27,29-43], three of them in the femoral shaft [28,44,45], and only one fracture involved the condyle [46]. The treatment strategy has been described in 18 out of 20 reported femoral neck SF cases in the literature [23-27,29-36,38-40,42,43]. To our knowledge, seven out of 18 cases were treated conservatively [23,24,31,32,34,35,39], whereas the remaining 11 cases were treated operatively via internal fixation and compression using a dynamic hip screw [25-27,29,30,33,36,38,40,42,43]. According to the extracted data from the abovementioned cases, specific details on the rehabilitation and follow-up were provided in 15 cases of long-distance runners. All of them successfully returned to their running routine following the post-injury management, within a period of eight weeks to two years [23-25,30,31,33,35,36,38-40,43-46]. It has been noted that two female runners were found underweight (BMI < 18.5 km/m^2^) [23,38]. According to Okamoto et al., one patient was further diagnosed with primary amenorrhea and eating disorders related to the female athlete triad [38]. In addition, the female long-distance runner presented by Dugowson et al. was also oligomenorrheic [28].

The conclusions we reach regarding the treatment and rehabilitation of femoral SFs indicate that the approach can vary depending on the specific case. In cases of femoral neck SFs, both conservative and operative treatments have been described in the literature. Conservative management involves rest, partial weight-bearing, and physical therapy interventions to promote healing and restore function. On the other hand, operative treatment options, such as internal fixation using a dynamic hip screw, have been employed in select cases to provide stability and facilitate faster healing. The decision between conservative and operative approaches depends on various factors, including the severity of the SF, the anatomical location, and patient characteristics. Following treatment, a gradual and supervised rehabilitation program is essential for long-distance runners to safely return to their pre-injury running routine. Physical therapy interventions play a significant role in the rehabilitation process, focusing on strengthening the surrounding muscles, improving gait mechanics, and gradually reintroducing weight-bearing activities. The rehabilitation process may vary in duration, ranging from several weeks to several months, depending on the severity of the SF and individual response to treatment.

Fibula

Although the lower extremity is the most common anatomical site for SFs to occur in long-distance runners, the vast majority of injuries occur in the tibia, therefore the fibula involves only a few reported cases [47]. This is probably due to greater mechanical loads applied on the tibia compared to the fibula during weight bearing [47]. Subsequently, the fibula receives clearly reduced loads and SFs are less frequent on that bone. The fracture may involve the entire length of the bone; however, it most commonly occurs in the middle and distal third of the fibula [47].

Reviewing the literature, we only found one case regarding SF in a marathon runner [47]. According to the only presented case in the literature by Lacroix et al., the patient was a 25-year-old male who suffered a very rare type of fibula neck SF during a marathon race [47]. He was treated conservatively for six weeks and he had a successful return to his running training routine.

Treatment and rehabilitation of fibula SFs typically involve a conservative approach due to the reduced mechanical loads and less frequent occurrence of SFs in this bone. Conservative management may include a period of rest, partial weight-bearing, and the use of assistive devices. While there is limited literature available specifically addressing fibula SFs in long-distance runners, the successful outcome of conservative treatment reported in the identified case suggests that a similar approach may be effective in promoting healing and enabling a safe return to running activities.

Foot and Ankle

Foot SFs may occur after repeated strain on every single bone of the foot. However, the metatarsal and tarsal bones as well as the navicular and sesamoid bones are the most common anatomic sites involved in the foot [48]. Typically, foot SFs are well-controlled with a conservative treatment [48].

Based on the literature, nine long-distance runners with a mean age of 21.8 ± 8.4 (from 16 to 38) years suffered a foot SF [48-54]. Three of them were male [50,53,54], while six of them were female [48,49,51,52]. Four out of nine cases included navicular bone [50,51], two of them included metatarsal bones [52,53], and the remaining three cases included the sesamoid [54], cuboid [49], and tarsal bones, respectively [48]. Six of the cases were treated conservatively [49-52,54], while two of them were treated operatively [48,53]. The duration of rehabilitation and return to sport in seven out of nine cases that provided data varied from eight weeks to one year after the onset of symptoms [48-53]. It is notable that two cases were suffering from iron deficiency anemia [48,49]. One of them (Kubo et al.) was diagnosed with amenorrhea and was underweight [48]. In addition, one case presented by Bean et al. had a past medical history of anorexia nervosa [49]. Subsequently, both of the aforementioned female long-distance runners were suffering from the female athlete triad. Moreover, two out of nine cases reported a history of previous SF on foot [49] and tibia [51], respectively. Of particular note are the cases presented by Murray et al. where two young female oligomenorrheic identical twins presented with a similar SF injury within a three-week period, after following a similar training plan [51].

The treatment and rehabilitation of foot SFs primarily involve conservative measures, as reported in the literature retrieved. Conservative treatment options for foot SFs may include rest, activity modification, immobilization with a cast or walking boot, and the use of assistive devices for weight-bearing as well as physical therapy interventions. The duration of rehabilitation and return to sport can vary depending on the severity of the SF and individual factors. Close monitoring and follow-up are essential to ensure proper healing and prevent complications or reinjury. In cases where operative intervention is deemed necessary, surgical options such as internal fixation may be considered, but such instances appear to be less common in foot SFs. Overall, the literature suggests that conservative management is generally effective in promoting healing and enabling a successful return to running activities for long-distance runners with foot SFs.

Pelvis

As previously mentioned, the lower extremity is the most common site for SFs to occur; however, a pelvic SF is an uncommon injury in long-distance runners [55]. They usually appear in the pubic ramus near symphysis, but SFs at the sacrum, the acetabulum, and the iliac bone have also been reported [55]. They are commonly presented as lower back or groin pain. The vast majority of pelvic SFs undergo conservative treatment, due to their classification as low-morbidity fractures [55].

Based on our literature review, 13 articles presenting pelvis SFs were identified, representing a total of 31 cases of long-distance runners suffering from a pelvic SF with a range between 18 and 58 years old [3,55-66]. Twenty-one out of 31 were female athletes [55,57-61,63-66] and few of them were elite female marathon runners. Sixteen cases were diagnosed with SF at the pubic ramus [59,63], nine cases were experiencing a sacrum SF [56,58,61,64-66], five cases had an iliac bone SF [55,57,60,62], and one with an acetabular SF [3]. According to Noakes et al., all seven out of seven cases presenting had a positive standing test [59]. All cases with pelvis SF were treated conservatively. Follow-up management was reported in 19 out of 31 cases, with all runners returning to their previous activity within a period of four weeks to one year [55-61,64-66].

As supported by the case reports of the literature retrieved, the treatment and rehabilitation of pelvic SFs in long-distance runners predominantly involve conservative management. Due to their classification as low-morbidity fractures, pelvic SFs are commonly treated without surgical intervention. Conservative treatment measures may include activity modification, rest, and targeted physical therapy interventions. Close monitoring and follow-up are crucial to ensure adequate healing and a successful return to running activities. The duration of rehabilitation and return to sport can vary depending on the severity of the SF and individual factors. The available literature indicates that conservative treatment for pelvic SFs in long-distance runners has demonstrated positive outcomes, with runners typically resuming their previous level of activity within a range of four weeks to one year.

Patella

Due to the rarity of patellar SFs, as well as the variable differential diagnoses of anterior knee pain in long-distance runners, the diagnosis of a patella SF is difficult to establish. Patellar tendonitis is a common clinical finding that mimics the symptoms and the history of a patella SF [67]. Patella SFs can be either longitudinal or transverse [68].

Three patella SF cases involving two male and one female long-distance runner are presented in recent literature [67,69,70]. The mean age of the three injured athletes was 39.7 ± 17.3 years [25,30] and 64 years old, respectively. It is notable that two of them had bilateral patella SFs [67,69], while the third one had a longitudinal SF [70]. The article by Thompson et al. presented a female elite Olympic marathon runner who was treated operatively following a non-sufficient conservative treatment of a one-sided patella SF. Interestingly, the immediate return to the training routine caused a new SF in the contralateral patella as well, which was initially treated surgically [69]. Another long-distance runner sustained bilateral patella SFs. The first one, a transverse fracture, was treated operatively while the longitudinal SF was treated conservatively [67]. All patients turned to their previous running activity level with excellent outcomes [67,69,70].

In conclusion, the treatment and rehabilitation of patellar SFs in long-distance runners require careful consideration. Due to the challenging nature of diagnosing patella SFs, it is important to differentiate them from other conditions. Treatment approaches may vary depending on the severity and characteristics of the SF. Operative intervention may be necessary in cases where conservative treatment fails or in specific situations, such as bilateral SFs or insufficient response to non-surgical methods. However, conservative management can often yield positive outcomes for patella SFs, allowing runners to return to their previous activity levels. Tailored rehabilitation programs, including physical therapy and gradual progression of exercise, are crucial for promoting proper healing, reducing the risk of re-injury, and facilitating a successful return to running. Close monitoring and follow-up are essential to ensure optimal recovery and long-term athletic performance.

Discussion

**Figure 2:**
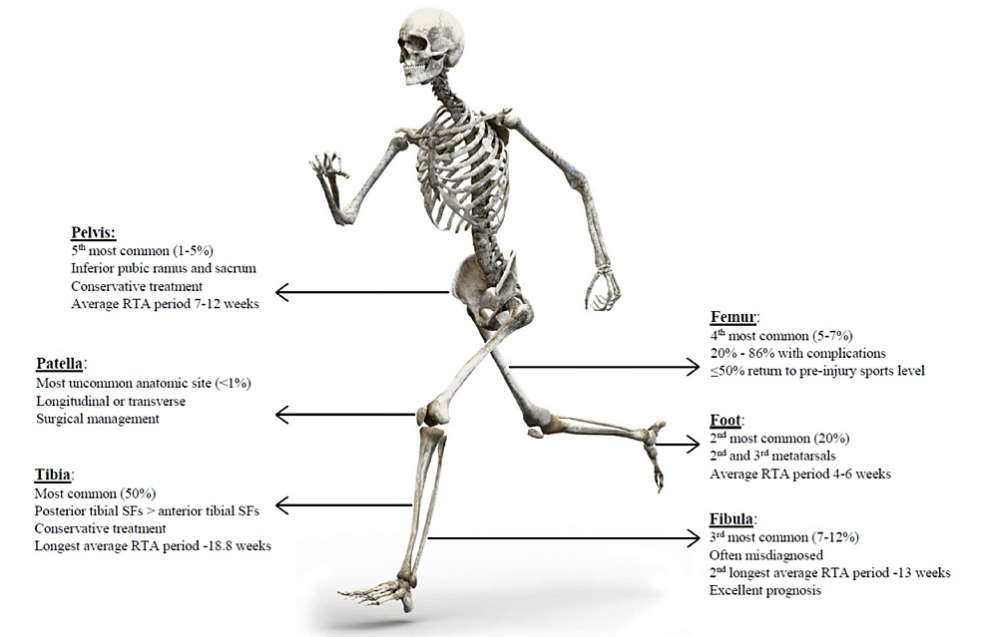
Schematic presentation of stress fractures per anatomic site. RTA: return to activity. Image source: https://www.pixabay.com (graphics and data available for editorial use under the Creative Commons license). The figure was edited by the first author (SH) for this study.

Bone SFs are the result of continuous microscopic fractures caused by recurring loads over the osteoblastic activity [71]. SFs are a frequent injury among the long-distance runner community and should remain a source of concern among runners as well as clinicians, due to their treatment-specific features and their high incidence of recurrence. Based on the present literature review, the most common anatomical site of this fracture type is the tibia [3]. However, most literature case reports represent femur and pelvis SFs. This is probably explained by the greater number of treatment complications as well as the longest rehabilitation period of these SFs [23-46,55-66]. Informative statistics and the return time to activity (RTA) for all reported SFs per anatomic site are represented in Figure 2.

SFs can be generally characterized as an overuse injury [1]. Most of the aforementioned injuries occurred when the athletes exceeded their normal training volume or intensity (“too much too soon” scenario) or changed the conditions under which the training took place. These conditions represent the extrinsic risk factors of an SF [72]. Nevertheless, insufficient bone health could also be a reliable cause for SF, representing the intrinsic risk factors of this injury. The association between a fracture and the female athlete triad, encompassing menstrual dysfunction, low energy availability, and decreased bone mineral density, is noteworthy, especially given its prevalence in women. Addressing this triad helps improve muscle balance, preventing the development of SFs and preserving high-level competition performance [6,7]. Additionally, two cases involving postmenopausal women were included, highlighting the predisposition of osteopenia and osteoporosis in SFs [24,57]. Furthermore, iron deficiency and anemia were identified as significant independent risk factors for SFs [48,49].

Of the 73 cases examined in this study, 35 patients were male [3,19-21,25-27,30,33,34,36,37,39,40,42-44,46,47,50,53,54,56,58,59,62,67,70] while 38 were female [23,24,28,29,31,32,35,38,41,45,48,49,51,52,55,57-61,63-66,69], showing a small propensity for SFs in female population. However, the literature shows a higher tendency of SFs in females compared to male long-distance runners [6,7]. A summary of all reported cases is shown in Table 1.

**Table 1:** Summary of the treatment management and rehabilitation of all cases reported in the literature. SF: stress fracture; M: male; F: female.

Author	Age	Sex	Type of fracture	Treatment	Rehabilitation (weeks)
Tibia					
Graham et al. (2018) [21]	32	M	Proximal tibial SF	Conservative	-
Bargfeldt et al. (2011) [20]	23	M	Posterior tibial shaft SF	Conservative	9
Saifuddin et al. (1994) [19]	21	M	Longitudinal tibial SF	Conservative	-
Vossinakis et al. (2000) [17]	38	M	Medial tibial condyle SF	Conservative	-
Daffner et al. (1982) [18]	15	M	Proximal tibial SF	Conservative	-
Femur					
An et al. (2017) [24]	64	F	Subchondral insufficiency fracture of the knee	Conservative	130
Baer et al. (1984) [25]	36	M	Femoral neck complete, undisplaced SF	Operative	12
Cichy et al. (2010) [26]	23	M	Femoral neck minimally displaced SF	Operative	-
Clough et al. (2002) [27]	55	M	Femoral neck displaced SF	Operative	-
Dugowson et al. (1991) [28]	32	F	Femoral shaft SF	Operative	-
Farkas et al. (2006) [29]	48	F	Subtrochanteric SF	Operative	-
Gerstmeyer et al. (2022) [30]	29	M	Femoral neck displaced SF	Operative	Returned after treatment
Jasqui-Remba et al. (2019) [31]	16	F	Bilateral femoral neck SF	Conservative	39
Kupke et al. (1993) [32]	29	F	Femoral neck compression SF	Conservative	-
Kerr et al. (1995) [33]	32	M	Femoral neck basicervical SF	Operative	35
Katsougrakis et al. (2016) [23]	28	F	Femoral neck compression SF	Conservative	8
Krause et al. (2018) [34]	38	M	Femoral neck compression SF	Conservative	-
Lamothe et al. (2018) [35]	23	F	Femoral neck SF	Conservative	26
Nadwodny et al. (2022) [36]	Young	M	Intertrochanteric SF	Operative	17
Nielsen et al. (2009) [37]	32	M	Femoral neck dislocated SF	-	-
Okamoto et al. (2010) [38]	17	F	Femoral neck displaced SF	Operative	104
Seki et al. (2016) [39]	16	M	Femoral neck compression SF	Conservative	13
Scott et al. (1999) [40]	50	M	Femoral neck SF	Operative	12
Sterling et al. (1993) [41]	42	F	Femoral neck SF	-	-
Taylor-Haas et al. (2011) [42]	34	M	Femoral neck SF	Conservative failed. Operative	-
Weinrich et al. (2021) [43]	38	M	Femoral neck complete, undisplaced SF	Operative	52
Weishaar et al. (2005) [44]	19	M	Femoral shaft SF	Conservative	12
Weind et al. (2005) [45]	15	F	Bilateral femoral shaft SF	Conservative	10
Yao et al. (2019) [46]	48	M	Femoral supracondylar SF	Conservative	104
Fibula					
Lacroix et al. (1992) [47]	25	M	Neck of the fibula SF	Conservative	6
Foot					
Kubo et al. (2019) [48]	18	F	Posterior talar process SF	Conservative failed. Operative	15
Bean et al. (2020) [49]	37	F	Cuboid SF	Conservative	52
Goergen et al. (1981) [50]	17	M	Tarsal navicular body complete SF	Conservative	39
	19	F	Navicular SF	-	-
Murray et al. (2005) [51]	17	F	Navicular SF	Conservative	8
	17	F	Navicular SF	Conservative	8
Shiraishi et al. (1993) [52]	17	F	Second metatarsal SF	Conservative	52
Percy et al. (1980) [53]	16	M	First metatarsal SF	Operative	30
Christiaans et al. (2004) [54]	38	M	Medial sesamoid bone SF	Conservative	-
Pelvis					
Alsobrook et al. (2007) [56]	36	M	Bilateral sacral SF	Conservative	7
Battaglia et al. (2011) [57]	58	F	Superomedial iliac SF	Conservative	7
Major et al. (2000) [58]	18-49	3M, 1F	Sacrum SF	Conservative	4
Noakes et al. (1985) [59]	34	M	Pubic ramus SF	Conservative	12
	30	F	Pubic ramus SF	Conservative	28
	21	F	Pubic ramus SF	Conservative	12
	40	M	Pubic ramus SF	Conservative	12
	21	F	Pubic ramus SF	Conservative	15
	34	M	Pubic ramus SF	Conservative	14
	36	M	Pubic ramus SF	Conservative	8
West et al. (2017) [60]	50	F	Iliac body SF	Conservative	5
Miletic et al. (2012) [61]	21	F	Sacral wing SF	Conservative	26
Lizawa et al. (2021) [62]	49	M	Iliac wing SF	Conservative	-
Pavlov et al. (1982) [63]	19-48	9F	Pubic ramus SF	Conservative	-
Klossner et al. (2000) [64]	19	F	Sacral ala SF	Conservative	39
Garcia et al. (2020) [3]	51	M	Nondisplaced transverse acetabular SF	Conservative	-
Haun et al. (2007) [65]	26	F	Sacral ala SF	Conservative	52
Hameed et al. (2011) [66]	43	F	Sacral ala SF	Conservative	17
Vitale et al. (2018) [55]	35	F	Incomplete medial iliac SF	Conservative	17
	36	F	Nondisplaced superomedial iliac SF	Conservative	-
Patella					
Thompson et al. (2022) [69]	30	F	Bilateral patella SF	Operative	3
Carneiro et al. (2006) [67]	64	M	Bilateral patella SF	Operative	52
Dickoff-Hoffman (1987) [70]	25	M	Longitudinal patella SF	Conservative	13

Although there is no definitive healing and rehabilitation period, it usually varies between four and 12 weeks [71]. From the cases studied above, the pelvis shows the lowest RTA time (3.5 months approximately) while the femur shows the greatest (10 months approximately). This is probably explained by the fact that the majority of pelvic SFs were treated conservatively, while most femoral neck SFs were treated by internal fixation and a dynamic hip screw [23-46,55-66]. In addition, delayed treatment has been correlated with prolonged return to activity time and therefore outlines the importance of an early SF diagnosis [71].

Prevention of SF in runners is a great challenge. Early identification and regulation of extrinsic and intrinsic risk factors is required. Extrinsic risk factors could be characterized as factors that modify the loads applied to a bone. Modifying extrinsic risk factors can be applied by controlling training factors (avoiding extreme changes in running speed, duration, and frequency), muscle factors (increased muscle mass decreases the transmission of loads across the kinetic chain), and running surface conditions as well as shoes and inserts (attenuating ground impact forces). Intrinsic risk factors could be described as the factors that modify the ability of a bone to resist load without damage accumulation. These factors include physical activity history and energy availability, as well as vitamin D and calcium levels, and their control could prevent SFs in runners. Finally, biomechanical factors that could have intrinsic (abnormal kinetic chain motion) or extrinsic (abnormal forces) influence on the bones have to be predicted as early as possible to avoid SFs in long-distance runners [73,74].

The lack of scientific assessment and the predominance of case reports in the context of SFs among long-distance runners is a noteworthy topic of discussion. While case reports provide valuable insights into individual experiences, they may not always capture the broader picture or establish causal relationships. Consequently, there is a significant gap in the existing literature, as no comprehensive review has been published on this specific topic. Therefore, the purpose of this scientific paper-article is to address this gap and provide a comprehensive review of SFs in long-distance runners. By synthesizing available case reports and existing knowledge, this review aims to shed light on the treatment and rehabilitation of SFs in this population. It also emphasizes the urgent need for rigorous scientific studies, including larger sample sizes, randomized controlled trials, and longitudinal investigations. Such research endeavors would significantly contribute to our understanding of SFs among long-distance runners and facilitate the development of evidence-based prevention strategies and tailored interventions for effective treatment and rehabilitation.

Strengths and limitations

To our knowledge, the present article is the first to review the treatment management and rehabilitation of SFs among long-distance and marathon runners. In addition, the extracted data from published case reports and case series were well categorized and presented per anatomic site where SFs are observed. In our point of view, the present study clarifies the treatment strategy followed in several cases and summarizes the return to sport expectations among long-distance runners. Nevertheless, our study’s limitation is that a large number of published cases did not include follow-up observations, therefore secure details on the return time to a high-level performance were not exactly estimated.

## Conclusions

In conclusion, SF is a clinical entity that affects a large number of athletes, especially long-distance runners. This study was conducted to identify the background of the injury among long-distance runners, as well as the management and prognosis of this type of fracture. As the running community has grown rapidly over the past few years, SFs are becoming a more common occurrence in emergency departments across the world. A high degree of clinical suspicion must be carried by the clinicians, including SFs, in the differential diagnosis of long-distance runners complaining of chronic musculoskeletal pain. The main aim is prevention, early diagnosis, and early treatment of SFs in long-distance runners so that return to high-level athletic performance may be achieved as soon as possible.
